# Vehicle re-identification based on dimensional decoupling strategy and non-local relations

**DOI:** 10.1371/journal.pone.0291047

**Published:** 2024-01-02

**Authors:** Xi Li, Xiyu Pang, Qinglan Meng

**Affiliations:** School of Information Science and Electrical Engineering, Shandong Jiaotong University, Jinan, Shan Dong, China; Hainan Normal University, CHINA

## Abstract

Vehicle re-identification (Re-ID) is a challenging task that aims to recognize the same vehicle across different non-overlapping cameras. Existing attention mechanism-based methods for vehicle Re-ID often suffer from significant intra-class variation and inter-class variation due to various factors such as illumination, occlusion, viewpoint, etc. In this paper, we propose a novel network architecture for vehicle Re-ID, named Dimensional Decoupling Strategy and Non-local Relationship Network (DMNR-Net), which uses three modules to extract complementary features: global feature extraction module, non-local relationship capture module(NRCM), and dimensional decoupling module (DDS). The global feature extraction module captures complete and coarse-grained features from the whole image; the NRCM module extracts saliency information from feature maps in both spatial and channel dimensions; and the DDS decouples spatial and channel features into two branches to extract fine-grained features and focus on specific subspaces. We conduct extensive experiments on two popular publicly datasets, VeRi-776 and VehicleID, to evaluate the effectiveness of our method. The experimental results show that our DMNR-Net outperforms state-of-the-art methods by a large margin on both datasets.

## 1. Introduction

The purpose of vehicle re-identification (Re-ID) is to recognize the same vehicle from non-overlapping camera views. While camera-based license plate identification technology [1–3] is utilized for auxiliary vehicle identification in some scenarios, it faces numerous challenges in real traffic environments. Factors such as multiple viewing angles, varying lighting conditions, and inconsistent camera resolution can significantly impact the accuracy of license plate identification. Additionally, license plates may be obstructed, decorated, forged, or removed, rendering it difficult to obtain clear license plate information.

Recently, with the rise of neural networks and the proposal of large-scale datasets. Some methods use external resources [4–7] to capture discriminative features such as: part resolution, pose estimation, foreground segmentation, vehicle view labeling, etc. PVEN [4] uses a vehicle part parser to generate four different view masks, and then generates aligned local features by averaging pooling of the masks. Miao et al. [6] learn part features through pose estimation bootstrapping and train part visibility through pseudo-labels generated by graph matching. Although these approaches using additional models or semantic labels achieve excellent performance, they all require additional data annotation and model training. This is not only labor-intensive and time-consuming for annotation, but also increases the computational complexity of the vehicle Re-ID task. To address the problems with the above methods, some other methods [8–13] focus on mining fine-grained cues in local regions using rigid divisions of predefined parts. HSKT [8] uses a rigid segmentation strategy to segment the vehicle image into multiple parts, and then directly uses these parts to extract local features. Chen et al. [9] divides the feature maps of vehicle images along different directions to extract rich fine-grained local features. Although these methods are simple and effective, the information captured between different parts can interfere with each other, so we propose a dimensional decoupling strategy that allows different parts to focus only on themselves, thus improving the characterization of features.

**Fig 1:**
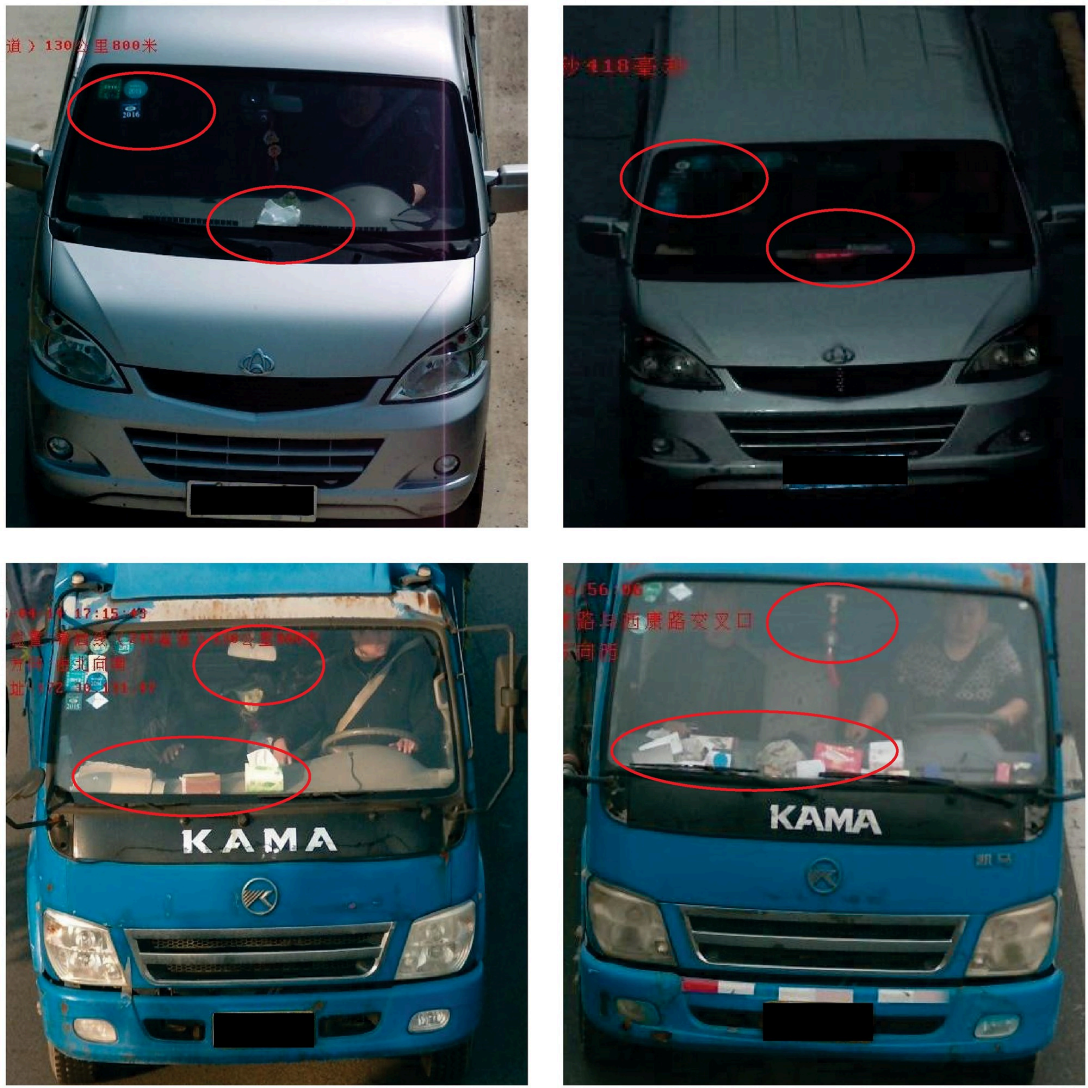
Each row is composed of the same set of two different vehicles, and these images are derived from the Veri-776 dataset. The details used to distinguish different vehicles are marked with red circles. It can be observed that their differences mainly lie in some subtle features. For example, the two different vehicles in the first row differ in terms of annual inspection marks and Interior trims; The second line is extremely similar in appearance to two different vehicles, with the difference being in the middle net decoration and interior fittings.

In addition, the attention mechanism is derived from the human visual information processing mechanism, which focuses on the information in the image that is useful for identity identification and filters out the information that is not useful for identity identification by means of an adaptive weighting. To better distinguish vehicles with similar appearance in Fig 1, the problem of small inter-class differences in vehicle Re-ID task is solved. Many studies [14–20] have shown the importance of applying attention mechanisms to the vehicle Re-ID task. DGPM [14] introduced non-local attention [15] after dividing the feature map into multiple regions using rigid division to enhance attentional modeling. CBAM [16] proposes a spatial attention module and a channel attention module that can focus on where the important features are in the spatial dimension and on which features are important in the channel dimension. TBE-Net [20] proposes a three-branch embedding network with part-aware ability and feature complementary learning to enhance vehicle feature representation. Among them, in order to accommodate the changing vehicle appearance under non-overlapping cameras, TBE-Net proposes a third branch, called the complementary branch. This branch is implemented in two steps. Firstly, a global maximum pooling (GMP) is applied to divide the feature map of the backbone network into four sub-regions. Secondly, two different pooling operations, global average pooling (GAP) and global maximum pooling (GMP) operations, are used to obtain multi-granularity features. Therefore, we propose a new non-local attention mechanism. The feature maps are first compressed along different dimensions, and then attention weights are explicitly inferred based on the compressed information, which not only enables the network to focus on the subject information of the target in the image to suppress complex background information using pairwise self-affinity between elements, but also reduces the computational cost of attention modeling.

In this paper, we propose a non-local relationship capture mechanism. Since the global context information of the input feature map is not always useful, only specific non-local relations play a significant role in improving the network accuracy; instead, the redundant non-local relations can become an interfering factor for the network to capture discriminative features. The redundant non-local relations not only occupy computational resources but also interfere with the network’s ability to capture real discriminative features. This reduces the accuracy of the vehicle Re-ID network. Therefore, we propose the non-local relation capture module to overcome difficulties of excessive computation caused by the existing methods by calculating all relations between each location. Based on this mechanism, we design two non-local relationship capture modules with different dimensions: a channel based non-local relationship capture module (C-NRCM) and a spatial based non-local relationship capture module (S-NRCM). Specifically, in the spatial non-local relation capture module, we perform noise reduction operations on spatial-level non-local relations while assigning appropriate weights to them. Both modules use the number of such non-local relations as tunable hyperparameters for selecting the most useful parameters for improving network accuracy.

Finally, we propose a dimensional decoupling strategy. This strategy can solve the problem that the weight information between the partition channels interferes with each other when using the hardening score strategy to extract multi-granularity features. The dimensional decoupling strategy eliminates mutual interference between the partition channels and the spatial features in the vehicle re-identification network. It decouples the spatial features from the partition channels by using different convolution kernels for each channel. In addition, the dimensional decoupling scheme is a more accurate way to capture features compared to the hardened score scheme. The dimensional decoupling scheme can not only reduce most of the redundant computations in the hard partitioning scheme, but also effectively break the network accuracy bottleneck caused by the shortcomings of the hard partitioning scheme. We apply the proposed modules and strategy to a three-branch network to perform efficient vehicle Re-ID.

The main contributions of this paper are summarized as follows:

1. In order to enhance the feature representation, we propose a dimensional decoupling strategy. First, we divide the feature map into two parts in spatial dimension along the horizontal direction, and then we perform the decoupling operation along the channel dimension.
2. We design a novel non-local attention mechanism that not only enables the network to focus on the subject information of the target in the image to suppress the complex background information using pairwise self-affinity between elements, but also reduces the computational cost of attention modeling. Based on the non-local attention mechanism, we design two attention modules (S-NRCM and C-NRCM) capable of explicitly inferring attention weights from the spatial dimension and channel dimension.
3. Extensive experiments on two popular public datasets show that our proposed method outperforms most existing methods.

## 2. Related work

### 2.1 Vehicle Re-ID

With the development of deep learning, feature learning using deep networks has become a common method for vehicle Re-ID. Vehicle Re-ID is similar to person Re-ID in that both suffer from the problem of small inter-class differences, but different vehicles belonging to the same manufacturer and model may have extremely similar appearances, and the challenge of small inter-class differences is more prominent in vehicle Re-ID. Some methods utilize external resources [4–7, 21, 22] and achieve higher performance than manual methods. However, they all require additional data annotation and model training. For example, DAReID [21] locates person parts by performing pose estimation on the image, and also performs feature extraction based on the key points obtained from pose estimation. He et al. [22] introduce a target detection network with ROI (region of interest) for each vehicle part, and then project the ROI into the global feature map generated by the global module to capture local information.

Meantime, the extraction of local information representing different features from different parts of the vehicle using a rigid division of predefined parts has been shown in many works [8–13, 23] to be an effective method to improve the Re-ID performance. MGN [13] divides the feature map into multiple stripes along the horizontal direction. However, the interference of related information between parts can make the effectiveness of these methods significantly limited. To enhance the feature representation, we propose a dimensional decoupling strategy to focus different part features on specific regions, which is used to strip the mutual interference of information between parts.

### 2.2 Attention mechanism

Recently, attention mechanisms [14, 16–19, 24] have been widely applied in the field of Re-ID to improve the performance of the model, which focuses on the information in the image that is useful for identity identification and filters out the information that is not useful for identity identification by means of an adaptive weight adjustment. AGNet [17] proposes an attention module to generate attribute masks can extract more discriminative features for category recognition. Self-attention [15, 25–32] as a special attention mechanism can refine the representation of each element by aggregating the features of all elements in a single sample based on pairwise self-affinity between elements. Excellent results have been achieved in various tasks such as image classification [26, 27], target detection [28, 29] and semantic segmentation [30, 31]. ViT [32] divides the input image into multiple patches of the same size and transforms them into tokens to perform the self-attention computation. TransReid [25] builds a strong baseline network based on self-attention to improve the accuracy of person Re-ID. In this paper, our proposed non-local relationship capture mechanism compresses the feature map along different dimensions and then explicitly infers attention weights based on the compressed information, which not only enables the network to focus on the subject information of the target in the image to suppress complex background information by using pairwise self-affinity between elements, but also reduces the computational cost of attention modeling.

## 3. Method

### 3.1 Network structure

**Fig 2:**
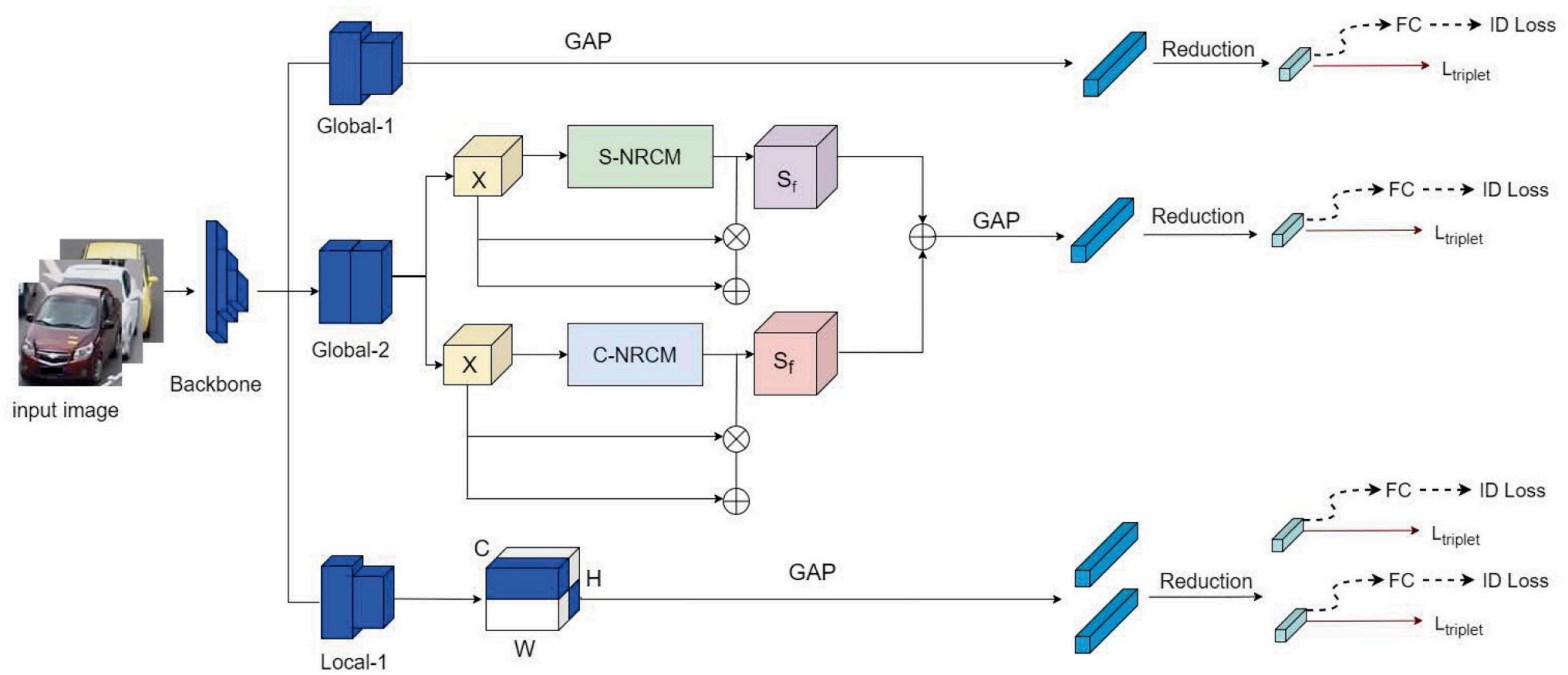
Network architecture of the DMNR-Net. The network includes three branches: Global-1, Global-2 and Local-1. GAP represents the global average pooling. Reduction represents the dimensionality reduction operation. FC represents the fully connected layer. *L*_*triple*_ and ID Loss represent the triplet loss function and the cross-entropy loss function, respectively. ⊗ indicates matrix multiplication. ⊙ indicates the element-wise multiplication operation between vectors, and ⊕ indicates element-wise summation. C-NRCM is a channel based non-local relationship capture module and S-NRCM is a spatial based non-local relationship capture module. During the network testing phase, the feature vectors from all branches are connected to form the final feature representation of the input image.

In this paper, we propose a three-branch network based on a dimensional decoupling strategy and non-local relations for vehicle re-identification. Some methods [13] use ResNet50 [33] and have shown excellent performance in image classification and target detection task. Therefore, we employ ResNet50 as the backbone network, which the network structure is illustrated in Fig 2. Similar to MGN [13], we split the part after the res_conv4_1 block into three separate branches that share a similar architecture with the original ResNet50 backbone. The first branch is used to extract the global features of images and is called Global-1. In Global-1, we utilized a stride 2 down-sampling operation on the residual block res_conv5_1 and applied global max pooling [34] on the corresponding output feature maps. Then we utilized a dimensionality reduction module consisting of a 1 × 1 convolution, batch normalization (BN), and an ReLU activation function to reduce the feature size from 2048 × 1 × 1 to 256 × 1 × 1. The Global-2 is designed using the non-local relationship capture mechanism proposed in this paper. In this branch, the tensor output from the res_conv5 block is first fed into both the spatial based non-local relationship capture module and the channel based non-local relationship capture module; i.e., a parallel structure is used to capture highly detailed spatial and channel correlations. This operation does not add much computational burden to the network. When keeping the feature size of the spatial and channel non-local relation capture modules unchanged, the next operation is the same as Global-1, which reduces the dimensionality from 2048 to 256 after global maximum pooling(GAP), a 1 × 1 convolution, batch normalization (BN), and ReLU operations.

Local-1 is our proposed dimensional decoupling strategy. At present stage, researchers use rigid division strategy to simply divide the input feature map at the spatial level, ignoring the feature information of each region in the channel dimension after performing rigid division. Because the features that need attention between each partition after rigid division are different, the region of importance in the channel dimension changes between each partition, and the research methods at this stage do not give a solution in this problem. We noticed this shortcoming during our research and adopted a more accurate feature capture method. A dimensional decoupling strategy for decoupling channels and spatial is proposed for the first time as the third branch of the vehicle re-identification network based on dimensional decoupling strategy and non-local relations.

The same operations are used in all three branches. First, the 256-dimensional features obtained by dimension reduction are used to calculate the triplet loss. Then, each 256-dimensional feature is fed into a fully connected layer to compute the cross-entropy loss. Finally, during the testing phase, all 256-dimensional features are concatenated together as the final feature representation to extract more discriminative information.

### 3.2 Spatial based non-local relationship capture module

**Fig 3:**
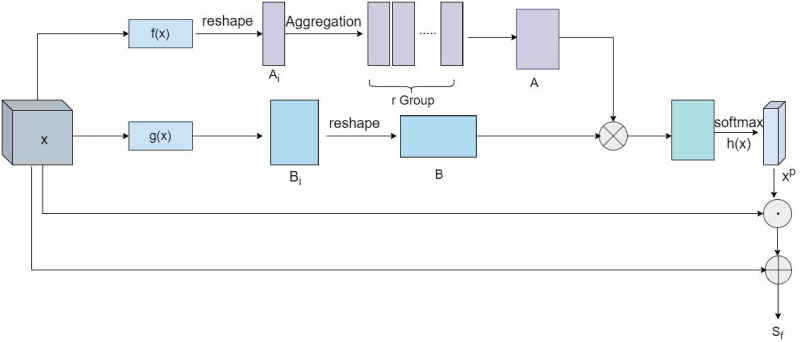
The architecture of the spatial based non-local relationship capture module (S-NRCM). f(*x*) and g(*x*) represent the two embedding functions. ⊗ indicates matrix multiplication. ⊙ indicates the element-wise multiplication operation between vectors and ⊕ indicates element-wise summation.

The S-NRCM aims to reduce the impact of noise by filtering out the appropriate global contextual information from the spatial level. As shown in Fig 3, *x* ∈ *R*^*H*×*W*×*C*^ is the input feature map of the S-NRCM, where *H* and *W* are the width and height of the input tensor, respectively, and *C* is the number of channels. In order to extract non-local information while reducing the computational effort, the feature map *x* is simultaneously passed through two embedding functions, f(*x*) and g(*x*), to obtain two matrices of different sizes *A*_*i*_ ∈ *R*^*C*×1×1^ and *B*_*i*_ ∈ *R*^*C*×*HW*^, where the function f(*x*) is composed of r deeply separable convolutions with a convolution kernel size of *HW*, and the function g(*x*) is composed of 1 × 1 convolution. In particular, we perform an r-group deep convolution operation on the feature map *x* to obtain an r-group global key distribution to measure the importance of each point, and because the size of the convolution kernel is *HW*, each set of convolutions fuses all features globally and assigns different weights to different positions. The r-group convolutions are used to represent the attention in the 1st, 2nd, 3rd, ……, r focus regions, where *r* is set as a tunable hyperparameter and the highest performance is selected by r times the rating weights. It is found experimentally that the best performance of this network is achieved when r = 2.

In addition, we deform the dimensions of the output feature maps *A*_*i*_ of size *C* × 1 × 1 for *r* groups to *C* × 1 and perform aggregation operations in the spatial dimension to obtain a matrix *A* = {*A*_1_, *A*_2_, …, *A*_*r*_} of size *C* × *r*. The matrix *B* of is obtained by a deformation operation on the matrix *B*_*i*_. Then, *A* is multiplied by *B* to obtain the non-local relationship matrix of size *HW* × *C*. In addition to the resulting spatial global context dependence, a *softmax* activation function is used for each column to obtain the appropriate probability matrix *x*^*p*^ = *R*^*HW*×*r*^. The process is expressed in the following equation:

$$\begin{array}{c} x^{p}=\text{softmax}(f(x)⊗g(x)) \end{array}$$ (1)

we perform an aggregation operation on each row of the probability matrix to obtain a weight vector *Q*. Each element *Q*_*i*_ of this vector represents the weight of the i-th spatial position, and the process is expressed in the following equation:

$$\begin{array}{c} Q_{i}=\sum_{j=1}^{r}x^{p}\left(i,j\right),i\in R^{HW} \end{array}$$ (2)

we use *h*(*x*) to represent the aggregation operation of each row of the probability matrix *x*^*p*^ to obtain the weight of each pixel. After broadcasting the size of *h*(*x*^*p*^) as *C* × *H* × *W* and multiplying it element by element with the original input feature map *x*, it is then added to *x* to obtain the final output feature *S*_*f*_ of the spatially based non-local relationship capture module. The process is expressed as follows:

$$\begin{array}{c} S_{f}=h\left(x^{p}\right)⊙x+x \end{array}$$ (3)

where ⊙ is multiplied element by element.

### 3.3 Channel based non-local relationship capture module

**Fig 4:**
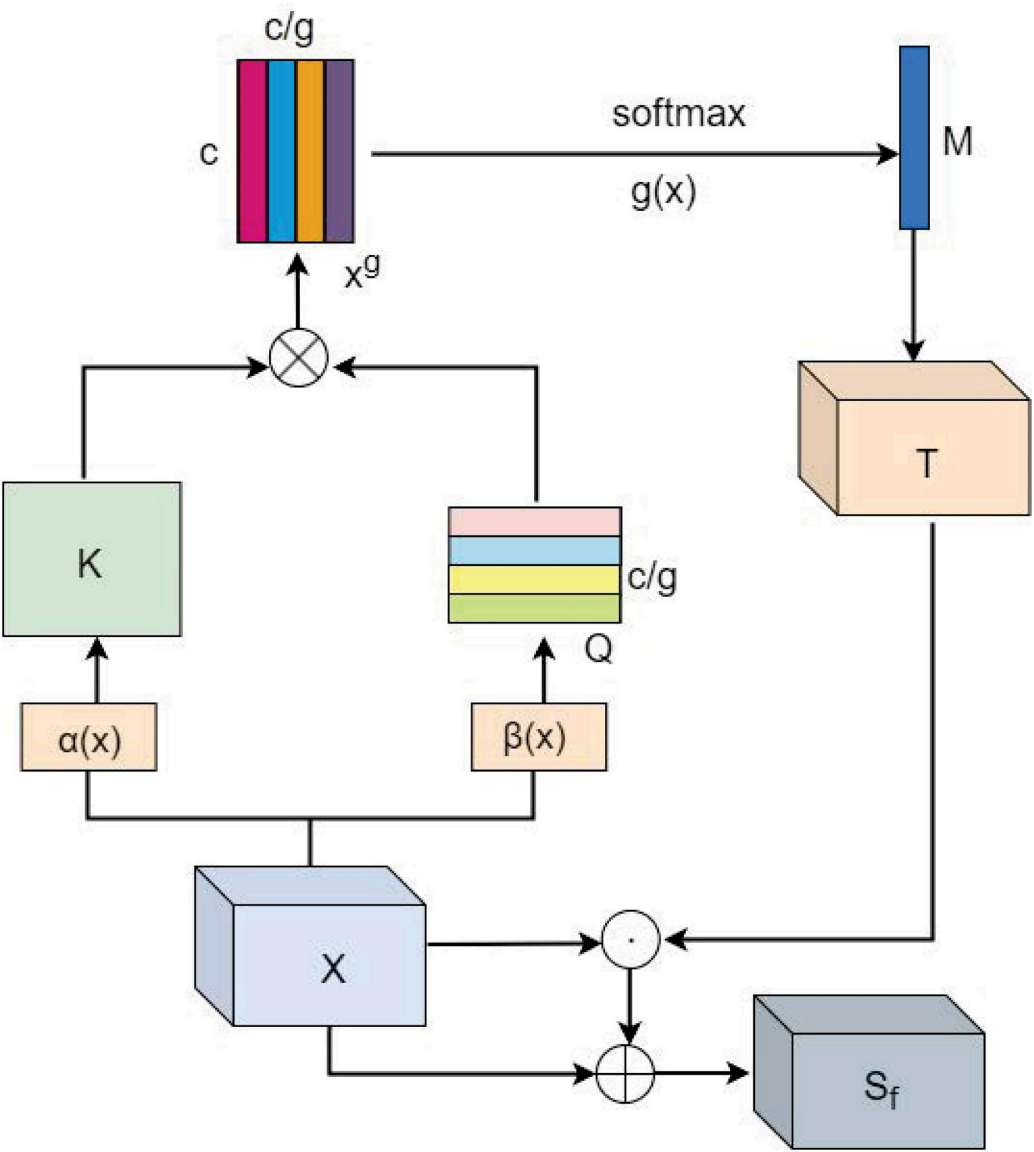
The architecture of the channel based non-local relationship capture module (C-NRCM). ⊗ indicates matrix multiplication, and ⊕ indicates element-wise summation.

The composition structure of the channel based non-local relationship capture module is shown in Fig 4. The original feature map *x* ∈ *R*^*H*×*W*×*C*^ is used as the input of the channel global context dependence module, where *C* represents the number of channels, *H* and *W* represent the height and width of the feature map, respectively. The channel attention module is used to construct global context dependencies for several groups of channels by modeling the relationships between channels to further improve the feature extraction capability.

First, we implement two convolution operations on the input feature map *x* to obtain the channel query matrix *Q* and the channel key matrix *K*. The sizes of the channel query matrix *Q* and the channel key moments *Q* are reshaped to (*HW* × (*C*/*g*)) and (*HW* × *C*), respectively. For the channel query matrix *Q*, since the number of parameters of grouped convolution is 1/*g* of the normal convolution, we use grouped convolution to compress its number of channels from *C* to *C*/*g* to reduce the computational effort. where *g* represents the channel compression parameter, and in the specific implementation to ensure the efficiency of the channel attention module, *g* is set to 4. Next, the global reference feature map *x*^*g*^ ∈ *R*^*C*×(*C*/*g*)^ is obtained by calculating the product of the matrices *Q* and *K*^*T*^. The computational equation of *x*^*g*^ is expressed as:

$$\begin{array}{c} x^{g}=Q⊗K^{T} \end{array}$$ (4)

where ⊗ denotes matrix multiplication. We use the *softmax* activation function for each column of *x*^*g*^ to obtain the probability matrix. The elements *x*_*i*,*j*_ at position (*i*, *j*) in the feature map *x*^*g*^ represent pairwise dependencies between the i-th channel and the j-th channel. The probability matrix is then fused in the row direction to obtain a global channel based feature mask M of size *C*×1. This operation aims to fuse channel pairwise dependencies to generate global contextual information about the channels. M is calculated as follows:

$$\begin{array}{c} M=\alpha (\text{softmax}\left(x^{g}\right)) \end{array}$$ (5)

where *α*(*x*) denotes the row fusion operation. Then, after broadcasting the size of M as *C* × *H* × *W*, it is multiplied element-by-element with the input feature *x* and then added with *x* to obtain the final output feature representation *S*_*f*_. The final output feature *S*_*f*_ ∈ *R*^*H*×*W*×*C*^ of C-NRCM is expressed as follows:

$$\begin{array}{c} S_{f}=M⊙x+x \end{array}$$ (6)

where ⊙ is multiplied element by element.

### 3.4 Dimension decoupling module

Some methods [8–12, 35] use a rigid division of predefined parts to extract local features of different parts. However, these methods simply divide the feature maps at the spatial level, ignoring the feature information of each region in the channel dimension after the rigid division. Therefore, we propose a dimensional decoupling strategy to decouple the spatial and channel dimensions, so that different part features focus on specific regions and strip the mutual interference of information between parts.

**Fig 5:**
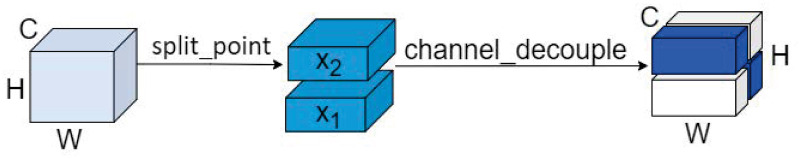
Diagram of the dimensional decoupling architecture. split_point indicates that the feature map is divided along the horizontal direction. channel_decouple indicates that the decoupling operation is performed in the channel direction.

When the image passes through the backbone network to get the input feature map *x* ∈ *R*^*C*×*H*×*W*^, where *C* is the number of channels, and *H* and *W* are the width and height of the feature map, respectively. First, we divide *x* into two parts in the spatial dimension along the horizontal direction to get two sub-feature maps *x*_1_ and *x*_2_.

$$\begin{array}{c} x_{1}=x[:{,}_{}:splitpo\text{int}{,}_{}:]x_{2}=x[:{,}_{}splitpo\text{int}{,}_{}:] \end{array}$$ (7)

where *splitpoint* = *H*/2. This extracts rich fine-grained local features, but like most existing methods, it leads to mutual interference between the divided part regions. To solve this problem, we perform a decoupling operation of *x*_1_ and *x*_2_ on the channels, which aims to excise the redundant channels in the channel dimension within the two spatial dimensions. As shown in Fig 5, we use the two white features in the feature map as the two subspaces after decoupling.

This not only reduces the computational load, but also strips the connection existing between spatial and channel. Compared with the previous method where each subspace is associated with all channels, the dimensional decoupling operation we designed truly allows local features to be extracted from only some channels and subspaces.

### 3.5 Loss function

#### Triplet loss function

Triplet loss function is a more widely used loss function in the current computer vision field. The neural network model based on Triplet loss can distinguish details well, and when two input images are extremely similar, the triplet loss can learn a better representation of these two less dissimilar input vectors and thus perform well in the classification task. Compared with other classification loss functions, triplet loss usually learns better fine-grained features in training. Therefore, we apply the triplet loss function to the 1024-dimensional feature vector of each branch for metric optimization of the network. At the same time, in order to meet the computational requirements of this loss, a small batch of P vehicles and K images of each vehicle is randomly selected from the training set. This loss function is defined as:

$$\begin{array}{c} L_{\text{tri}plet}=\sum_{m}^{P}\sum_{n}^{K}[\alpha +\max_{P=1,...,K}{\left\|X_{n}^{m}-X_{P}^{m}\right\|}_{2}-\min_{i=1,...,K,j=1,...,P,j\neq m}{\left\|X_{n}^{m}-X_{i}^{j}\right\|}_{2}{]}_{+} \end{array}$$ (8)

where $X_{n}^{m}$ is the feature extracted from the anchor, $X_{p}^{m}$ denotes features extracted from positive samples, and $X_{i}^{j}$ denotes features extracted from negative samples. *α* is the margin hyperparameter, which is used to increase the gap between the anchor and the positive sample pairs, as well as the anchor and the negative sample pairs. The notation [⋅]_+_ indicates max(⋅, 0).

#### Cross-entropy loss function

Cross-entropy loss [36] is a commonly used loss function in deep learning, which is usually used in classification problems. Vehicle Re-ID can be considered as a multi-classification problem, where each vehicle acts as a different class. Therefore, we apply the cross-entropy loss function to the vehicle Re-ID task. The essence of cross-entropy loss is to measure the distance between two probability distributions. One of the probability distributions is the distribution of the true labels, and the other probability distribution is the distribution predicted by the model. The smaller the cross-entropy value, the closer the two probability distributions are, and the better the model prediction is. The formula is as follows:

$$\begin{array}{c} L_{id}=\sum_{m=1}^{D}{-}^{}q_{m}\text{log}\left(p_{m}\right)\{{}_{q_{m}=1,x=m}^{q_{m}=0,x\neq m} \end{array}$$ (9)

where *D* represents the number of vehicle identities in the dataset, *x* is the true label of the ID, and *p*_*m*_ is the predicted probability of the ID for the mth class.

During network training, the total DMNR-Net loss is calculated as follows:

$$\begin{array}{c} L_{total}=L_{id}+L_{triplet} \end{array}$$ (10)

where *L*_*id*_ represents the cross-entropy loss function and *L*_*triplet*_ represents the batch hard triplet loss function.

## 4. Experiments

### 4.1 Experimental datasets and evaluation metrics

The VeRi-776 dataset is a commonly used benchmark dataset in the field of vehicle Re-ID. It consists of approximately 40,000 real-world vehicle images captured by twenty cameras at various angles and comprises three types of images: training, test, and query images. The training set comprises 11,579 images of vehicles captured from multiple angles, while the test set contains 37,778 images, including 1678 query images. The images were collected over five years in downtown Hong Kong and parking lots, showcasing diverse perspectives and lighting conditions, making it a highly representative and practical dataset. The VeRi-776 dataset, which provides a range of variations such as different scenes, lighting conditions, vehicle colors, and models, is widely used to address the cross-camera vehicle Re-ID problem. These variations pose a challenge to the algorithm’s robustness and generalization performance.

The VehicleID dataset is a large-scale image dataset that has been widely used in the field of vehicle Re-ID. It was released by the Laboratory of Computer Vision and Machine Learning at Renmin University of China and consists of 44,238 high-resolution images of vehicles captured at different times, angles, and locations in various cities across China, such as Beijing, Guangzhou, and Kunming. The dataset covers 110 different vehicle brands and models, including cars, SUVs, trucks, and vans, with each image containing a single vehicle. Due to the significant variations in appearance, color, and model among the vehicles, the dataset poses a challenging task. To facilitate data usage, the Vehicle ID dataset provides various annotation information, such as the brand, model, and color of the vehicles. Moreover, it offers multiple data partition methods, such as random partition and temporal partition, for effective training validation and testing.

Establishing an effective metric is crucial for accurately assessing the accuracy and generalization ability of a vehicle Re-ID network. This paper uses two widely employed evaluation metrics, mean average precision (mAP) and cumulative matching curve (CMC), to measure network performance. To overcome this limitation, the CMC curve is often paired with the mAP value to make an objective evaluation of the Re-ID network.

### 4.2 Experimental details

The backbone and branches of the DMNR-Net are initialized with pre-trained ResNet-50 weights trained on ImageNet [37]. To enhance performance, the input images are resized to 256 × 256 pixels and randomly horizontally flipped in the training set. The triplet loss function’s edge parameter is set to 1.2 for all experiments, and each mini-batch contains P vehicles with K images randomly selected from the training set to meet the criteria of the loss function. The optimizer of the proposed network is Adam with an initial learning rate of 0.002 that decays to 2e-4 and 2e-5 after 150 and 250 epochs, respectively. The training process lasts 450 epochs and the PyTorch framework is used to implement the model. To complete training on VeRi-776 dataset, it takes approximately 3.5–4 hours using data parallel acceleration on two NVIDIA 2080Ti GPUs. The Cumulative Match Characteristics (CMC) and mean average precision (mAP) are used to evaluate the proposed network’s accuracy and generalization ability.

### 4.3 Comparison experiment

In this paper, we compare DMNR-Net with other advanced methods. Tables 1 and 2 illustrate the findings of the experiments carried out on the VeRi-776 and Vehicle ID datasets, respectively.

**Table 1:** The results of DMNR-Net compared with the results of existing methods on VeRi-776.

Method	Rank-1	Rank-5	mAP
PCB [38]	0.944	0.969	0.686
DNN_CRF [39]	0.923	0.957	0.646
BFE [40]	0.840	0.910	0.666
DuATM [41]	0.902	0.964	0.676
FCN [42]	0.850	0.870	0.708
PVEN vs [43]	0.948	0.974	0.775
VCAM [44]	0.944	0.969	0.686
PGAN [45]	**0.965**	0.983	0.793
GMSI [46]	0.936	0.971	0.756
TCPM [47]	0.940	0.971	0.746
PRND [48]	0.943	0.987	0.743
LCDNet+BRL [49]	0.946	0.980	0.760
SN++ [50]	0.951	0.981	0.757
TBE-Net [20]	0.960	0.985	0.795
**DMNR-Net (Ours)**	0.958	**0.989**	**0.820**

**Table 2:** The results of DMNR-Net compared with existing methods on VehicleID.

Method	Small		Medium		Large	
	Rank-1	Rank-5	Rank-1	Rank-5	Rank-1	Rank-5
TAMR [51]	0.660	0.797	0.629	0.768	0.596	0.738
MSA [52]	0.776	0.905	0.744	0.863	0.729	0.846
RAM [53]	0.752	0.915	0.723	0.870	0.677	0.845
AAVER [54]	0.747	0.938	0.686	0.900	0.635	0.856
EALN [55]	0.751	0.881	0.718	0.839	0.693	0.814
PRN [56]	0.784	0.923	0.750	0.883	**0.742**	0.864
SN++ [50]	0.767	0.870	0.748	0.842	0.739	0.836
LRPT + TSAM + CP [49]	0.779	0.935	0.779	0.907	0.745	0.865
**DMNR-Net(Ours)**	**0.812**	**0.962**	**0.763**	**0.934**	0.735	**0.908**

#### Comparative analysis of experimental results on Veri-776

We compare the proposed model with other existing models on the VeRi-776 dataset, and the experimental results are shown in Table 1. It can be seen from the table that DMNR-Net achieves 82.0%, 95.8%, and 98.9% on mAP, Rank-1, and Rank-5 accuracies, which is better than most existing methods. First, Compared with TBE-Net [20], which also uses a three-branch network, we propose that DMNRNet can capture discriminative features in spatial and channel dimensions. It is 2.5% higher than TBE-Net in mAP.compared to VCAM [44] and FCN [42], which utilize only channel-based attention mechanisms, our proposed vehicle re-identification network based on dimensional decoupling and non-local relationships incorporates feature capture mechanisms in both the spatial and channel dimensions. As seen in Table 1, the mAP and Rank-1 accuracy of our method (DMNR-Net) are 82.0% and 95.8%, respectively. It is clear that the network that incorporates both spatial and channel non-local relationship capture mechanisms outperforms a network that incorporates only one-dimensional mechanisms.

Second, the multi-granularity feature representation in the PVEN vertical split (PVEN vs) model [43] uses U-Net to split the vehicle image into four horizontally divided parts, but this approach requires considerable labor and time to annotate the complex segmented parts. In contrast, our proposed method outperforms it by 4.5% and 1% in mAP and Rank-1 accuracies, respectively, and does not require any additional manual annotation. Finally, it is worth noting that although PGAN [45] achieves slightly higher (0.7%) than our method on Rank-1 accuracy, our method outperforms PGAN on both mAP and Rank-5 accuracy and reaches a performance improvement of 2.7% on mAP.

#### Comparative analysis of experimental results on VehicleID

Table 2 displays the experimental results of our method and other existing models across three different test datasets. We have highlighted superior performance in bold. To evaluate the effectiveness of our method, we have considered Rank-1 and Rank-5 accuracies, as each query vehicle only has one true positive. Across the three Vehicle ID test sets, we have reported Rank-1 accuracies of 81.2%, 76.3%, and 72.5%, and Rank-5 accuracies of 96.2%, 93.4%, and 90.8%, respectively. In larger test sets, our proposed network outperforms PRN [56] in all metrics except for Rank-1, where PRN’s performance is slightly better. This difference is likely due to PRN’s use of additional part labels and detection networks to pinpoint vehicle parts, which our network does not rely on.

### 4.4 Ablation study

To validate the network (DMNR-Net) and detail the important contributions of the two attention modules and dimensional decoupling branches proposed in the DMNR-Net to network performance improvement, we conducted a series of experiments on the VeRi-776 dataset to fully demonstrate the effectiveness of the key components of our approach. In this process, we choose the same structure with the Global-1 branch as a baseline network (Baseline).

#### Structural analysis of the NRCM module

In Table 3, The NRCM attention mechanism serves as the foundation for our design of the C-NRCM and S-NRCM. One crucial aspect that we consider is the spatial (channel) hyperparameters *r*(*g*), as they play a significant role in optimizing the structure and performance of the NRCM module.

**Table 3:** The hyperparameter r and g results on the test set of VeRi-776.

Method	mAP	Rank-1
Baseline	0.759	0.925
Baseline+Sab(r = hw/2)	0.803	0.952
Baseline+Sab(r = 2)	0.806	0.948
Baseline+Sab(r = 4)	0.802	0.946
Baseline+Cab(g = 4)	0.804	0.953
Baseline+Cab(g = 8)	0.801	0.949
Baseline+Sab(r = 2)+Cab(g = 4)	0.820	0.958

**(1)Choosing the optimal value for hyperparameter *r*.** The hyperparameter r represents the attention degree to the 1st, 2nd, 3rd, ……, r key regions. To ensure that the aggregation operation can capture all the global features and assign different weights to different positions, we typically set the hyperparameter r to values such as 2, 4, etc. To determine the value of hyperparameter r, we remove the C-NRCM and dimensional decoupling strategy from the DMNR-Net. The experimental results with different values of r are shown in Table 3. We can observe that the best results are achieved when the hyperparameter “r” in the spatial attention module is set to 2. It is worth noting that the second row of the Table 3,“Sab(r = hw/2)” indicates that the hyperparameter “r” in the spatial attention module is set to half the product of the height (h) and width (w) of the feature map, which is inferior to directly setting the parameter to “Sab(r = 2)”.

**(2)Choosing the optimal value for hyperparameter *g*.** In C-NRCM, we implement a channel dimension compression operation on the input feature map to improve the feature extraction ability while reducing the computational effort of the model. To verify the effectiveness of this operation in the channel module, we set the size of hyperparameter g to 4, 8, etc. According to the results in Table 3, we observe that “Cab(g = 4)” achieves the best accuracy on mAP, which indicates that it is effective for capturing global context dependencies. Table 3 shows that we achieve the best results when we set the hyperparameter “r” in the spatial attention module to 2 and the hyperparameter “g” in the channel attention module to 4.

#### The effectiveness of C-NRCM and S-NRCM

Table 4 presents the impact of the non-local relationship capturing attention modules on the network model’s performance in both the spatial and channel dimensions. “Baseline + sab” and “Baseline + cab” represent adding our designed spatial attention module and channel attention module to Baseline, respectively. Compared to “Baseline”, “Baseline + sab” improves mAP and Rank-1 accuracy by 0.61% and 0.58%, respectively, which demonstrates the effectiveness of the spatial attention module. Furthermore, compared to “Baseline + cab”, the channel level non-local relationship capturing module can increase mAP and Rank-1 accuracy by 0.45% and 0.52%, respectively, indicating the effectiveness of the channel-level non-local relationship capturing module. In addition, we a parallel structure for the channel-level and spatial-level non-local relationship capturing modules, and this structure is represented in Table 4 as “Baseline + sab + cab”. It can be observed that this method improves performance by 0.65% and 1.17% compared to “Baseline”. Overall, it can be concluded that embedding both modules can significantly enhance network performance, demonstrating the effectiveness of the spatial and channel attention modules.

**Table 4:** Experiments to verify C-NRCM and S-NRCM.

Method	mAP	Rank-1
Baseline	0.759	0.925
Baseline + sab	0.765	0.931
Baseline + cab	0.764	0.929
Baseline + sab + cab	0.771	0.932

#### Effectiveness of the dimensional decoupling strategy

Table 5 displays the outcomes of “Baseline+DDS-1” and “Baseline+DDS-2”, two distinct branch decoupling techniques. DDS-1 denotes the dimension decoupling mechanism in the two blue blocks of the third branch, as shown in Table 5, whereas DDS-2 refers to the two white blocks. Both methods, “Baseline+DDS-1” and “Baseline+DDS-2”, are effective in boosting network performance, with the latter approach exhibiting superior results.

**Table 5:** The dimensional decoupling strategy results.

Method	mAP	Rank-1
Baseline	0.759	0.925
Baseline +DDS-1	0.813	0.945
Baseline +DDS-2	0.815	0.952

#### Sorting visualization analysis

**Fig 6:**
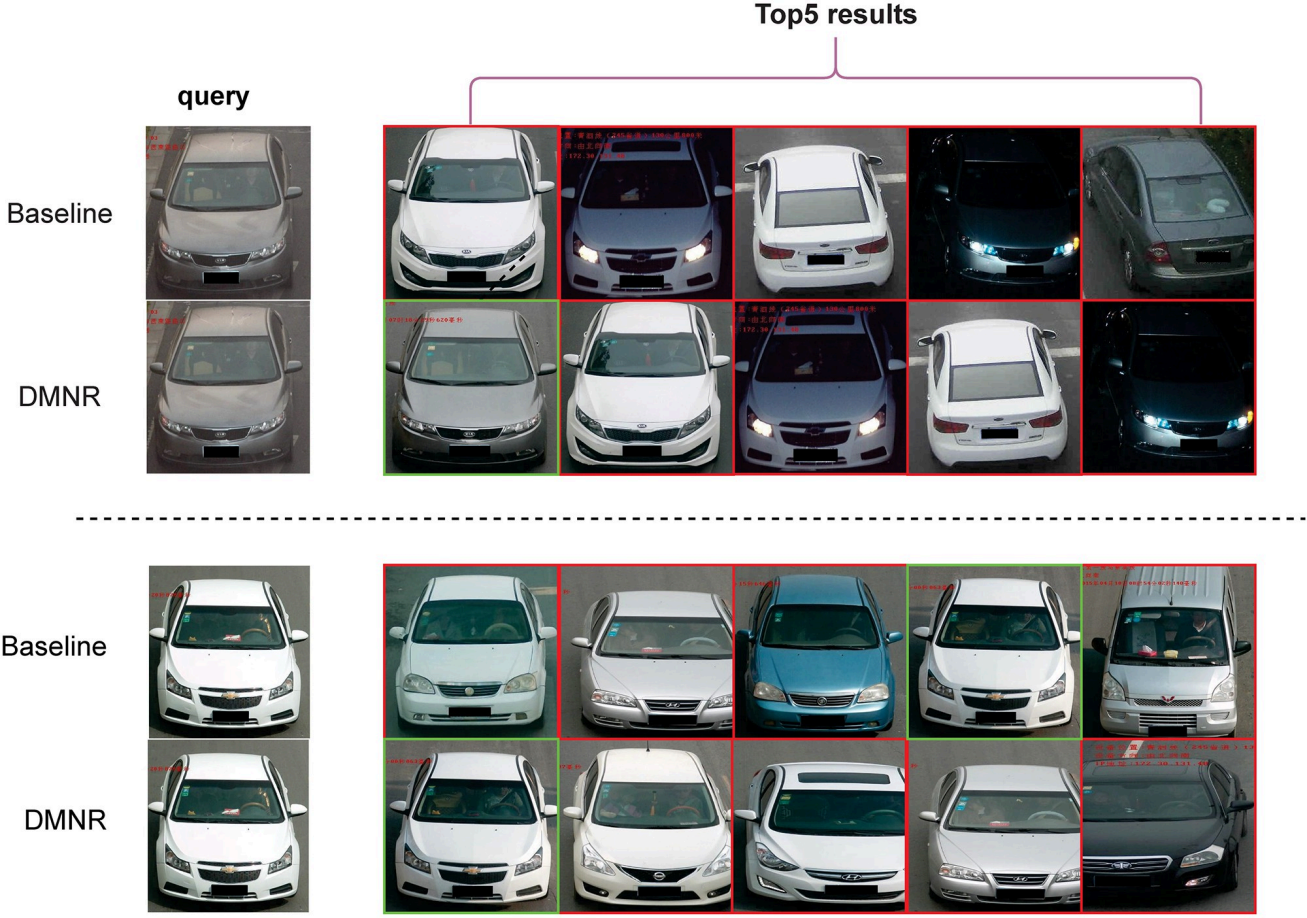
The vehicle retrieval results of each group query images, we generate a visualization of the ranking list on Vehicle ID dataset. The first column is the query image. the remaining columns are the top 5 vehicle images, which were retrieved from the test set. In all detection results, the images with green boxes and the query image are taken from the same vehicle, while images with red boxes are taken from different vehicles.

Fig 6 We performed a visual comparison of the ranking of Baseline and DMNR-Net on the Vehicle ID dataset to better demonstrate the effectiveness of vehicle re-identification networks based on dimensional decoupling strategy and non-local relationships. Retrieval results are shown in Fig 6. We can observe that the DMNR-Net can accurately identify the same vehicle identity even under different viewing angles and lighting conditions. This demonstrates the effectiveness of our proposed dimensional decoupling strategy and non-local relationships capture module in extracting highly robust vehicle features.

## 5. Conclusion

In this paper, a vehicle re-identification network based on dimensional decoupling and non-local relations (DMNR-Net) is proposed for the vehicle Re-ID task. The DMNR-Net consists of three branches that learn different types of discriminative representation information. The first branch (Global-1) captures more complete and coarse-grained features. For the second branch(Global-2), the feature maps obtained from the backbone network are enhanced with saliency information in both the spatial dimension and the channel dimension, which allows the network to extract more fine-grained features. The third branch(Local-1) completely decouples the spatial and channel dimensions so that a portion of the features are focused on a specific region. Overall, each of the three branches extracts different useful information, and the experimental results show that the three branches can assist each other in optimizing the performance of the model. The experiments conducted in this paper demonstrate that the proposed method achieves superior performance on popular datasets and surpasses the majority of existing methods.

Although our proposed method improves performance, the three-branch structure also introduces some computational complexity. In future work, we will investigate the effect of adding modules to a more lightweight network structure. Also, in many practical applications, vehicle Re-ID needs to be performed in a real-time environment. In future research, we will focus on how to design efficient algorithms and models to achieve fast and accurate vehicle Re-ID and to meet the real-time requirements.

## Supporting information

S1 File (ZIP)
